# Nicotinic Acetylcholine Receptors in Glial Cells as Molecular Target for Parkinson’s Disease

**DOI:** 10.3390/cells13060474

**Published:** 2024-03-07

**Authors:** Érica Novaes Soares, Ana Carla dos Santos Costa, Gabriel de Jesus Ferrolho, Rodrigo Portes Ureshino, Bruk Getachew, Silvia Lima Costa, Victor Diogenes Amaral da Silva, Yousef Tizabi

**Affiliations:** 1 Laboratory of Neurochemistry and Cell Biology, Department of Biochemistry and Biophysics, Institute of Health Sciences, Federal University of Bahia, Salvador 40110-902, BA, Brazil; 2 Laboratory of Neurosciences, Institute of Health Sciences, Federal University of Bahia, Salvador 40110-902, BA, Brazil; 3 Department of Biological Sciences, Universidade Federal de São Paulo, Diadema 09961-400, SP, Brazil; 4 Laboratory of Molecular and Translational Endocrinology, Escola Paulista de Medicina, Universidade Federal de São Paulo, São Paulo 04039-032, SP, Brazil; 5 Department of Pharmacology, College of Medicine, Howard University, 520 W Street NW, Washington, DC 20059, USA

**Keywords:** Parkinson’s disease, dopamine, acetylcholine, nicotine, nicotinic receptors, microglia, astroglia, oligodendrocyte, NG2 cells, alpha-synuclein, toll-like receptors, neuroinflammation, neuroprotection

## Abstract

Parkinson’s disease (PD) is a progressive neurodegenerative disease characterized by resting tremor, bradykinesia, rigidity, and postural instability that also includes non-motor symptoms such as mood dysregulation. Dopamine (DA) is the primary neurotransmitter involved in this disease, but cholinergic imbalance has also been implicated. Current intervention in PD is focused on replenishing central DA, which provides remarkable temporary symptomatic relief but does not address neuronal loss and the progression of the disease. It has been well established that neuronal nicotinic cholinergic receptors (nAChRs) can regulate DA release and that nicotine itself may have neuroprotective effects. Recent studies identified nAChRs in nonneuronal cell types, including glial cells, where they may regulate inflammatory responses. Given the crucial role of neuroinflammation in dopaminergic degeneration and the involvement of microglia and astrocytes in this response, glial nAChRs may provide a novel therapeutic target in the prevention and/or treatment of PD. In this review, following a brief discussion of PD, we focus on the role of glial cells and, specifically, their nAChRs in PD pathology and/or treatment.

## 1. Introduction

Parkinson’s disease (PD), where global epidemiological data show over 8.5 million individuals afflicted with it, is considered the second most common progressive neurodegenerative disorder [1]. Though many of the motor features of PD are dopamine (DA) responsive, some symptoms, including balance problems, do not respond well to such treatments. Non-motor symptoms such as cognitive difficulties, depression, fatigue, sleep problems, urinary problems, constipation, and variations in blood pressure do not respond to what might be termed as “DA-replacement therapy.” Thus, it is concluded that deficiencies in other neurotransmitter systems, including the nicotinic cholinergic system, may underlie these features [2,3,4]. As such, there is interest in targeting these other neurotransmitter functions to treat specific dopamine-resistant aspects of PD.

One of the challenges in PD is underscored by the fact that its pathogenesis is not totally clear. Epidemiological data show approximately 15% of PD patients have a hereditary form, and 5–10% have a monogenic mendelian inheritance that, so far, has 23 loci and 19 causative genes [5,6]. Moreover, mutations in the autosomal dominant genes, such as *SNCA, LRRK2,* and *VPS35*, or the autosomal recessive genes, such as *PRKN, PINK1*, and *DJ-1*, may lead to PD. On the other hand, recessive *DNAJC6* mutations can present as atypical Parkinsonism [7]. Additionally, the potential contribution of environmental toxins, such as herbicides (e.g., paraquat), insecticides (e.g., rotenone), an excess accumulation of iron or manganese [3,8,9,10], as well as exposure to endogenous toxins such as salsolinol or aminochrome, to PD pathology was verified [3,11,12].

Studies involving the hereditary form of PD, imaging, post-mortem, and extensive pre-clinical models implicated a set of molecular and cellular alterations. These include DAergic neuronal loss in the nigrostriatal pathway [13], mitochondrial damage [14], dysfunction in autophagy/mitophagy [15], the accumulation of alpha-synuclein aggregates [16], and neuroinflammation, underscored by gliosis [17]. Regarding the latter, it is of importance to note that in the brain, four main subsets of glial cells (microglia, astrocytes, oligodendrocytes, and synantocytes or NG2 cells) were identified. For each subset, the functional heterogeneity and beneficial or detrimental roles in maintaining homeostasis are under intense investigation.

Thus, given the important role of glial cells in the pathogenesis of PD [17,18,19,20] and the implicated nAChR regulation of these cells [17,18,21], our aim in this review is to provide not only an update on glial nAChRs’ role in glial function but also the potential exploitation of this knowledge in identifying novel targets for this devastating disease.

## 2. PD Pathophysiology and Current Treatments

The main pathological alterations in PD are Lewy body accumulation, composed principally of alpha-synuclein (α-Syn), and a loss of substantia nigra pars compacta (SNpc) DAergic neurons which leads to DA deficiency in the striatum [22,23,24]. This loss of DAergic neurons results in various motor deficits such as resting tremor, rigidity, akinesia, postural instability, and non-motor symptoms that may involve various other neurotransmitters [3,24,25]. Examples of non-motor symptoms may include emotional changes (e.g., depression, apathy, and anxiety), cognitive deficits (e.g., mild to severe memory impairment), autonomic dysfunction (e.g., bladder disturbances, orthostatic hypotension, sweating), gastrointestinal (GI) symptoms (e.g., constipation, nausea), sleep perturbations (e.g., insomnia/hypersomnia), and sensory symptoms (e.g., pain, visual and olfactory disturbances) [24,26,27]. DA replacement via L-dopa (L-3,4-dihydroxyphenylalanine), which is a DA precursor, is the most common treatment. However, not only the efficacy of this drug is invariably reduced in a few years, but long-term treatment may cause severe dyskinesia or involuntary movements [3,25,28,29]. Hence, extensive efforts to find novel therapies are ongoing.

The usual clinical treatment for PD aims to replace DA and inhibit the motor symptoms by using L-dopa or inhibitors of enzymes that metabolize DA, such as monoamine oxidase B (MAO-B) or catechol-O-methyltransferase (COMT) [30]. L-Dopa is usually combined with carbidopa to inhibit its peripheral metabolism. For stiffness and akinesia, L-dopa appears to be the best option as it improves the patient’s quality of life [24,31]; however, its chronic usage induces side effects, such as dyskinesia, motor fluctuations, and psychosis [24,32]. More recently, deep brain stimulation was employed for inhibiting motor symptoms with a relatively high effectiveness, although surgery-related complications remain a possibility [24,33]. Due to the limited efficacy and/or complications of current interventions, extensive effort, particularly towards the prevention of neurodegeneration, has been expended. In this regard, neuronal nAChRs have emerged as a viable target [4,34,35]. Interestingly, a mutation in RIC3, a chaperone of neuronal nicotinic acetylcholine receptor subunit α-7 (CHRNA7), was implicated in PD [7]. However, recent elucidation of the functional presence of nAChRs in nonneuronal cells (i.e., glia) may offer additional novel targets in preventing and/or slowing down the disease progression. The following delves into this possibility. 

## 3. Glial Cells

The glia represents the biggest population of cells in the human brain, with 10 times more cells than the neurons [36,37]. These cells perform several pivotal functions such as energetic support for neurons [38,39,40], the formation of the blood–brain barrier (BBB) [41,42], the regulation of neurotransmitters [43,44,45], the development and remodeling of synapses [46,47,48], detoxification [49,50,51], the control of the fluid/electrolyte homeostasis [52], the control of metabolism [53,54], neuroendocrine function [55], innate immunity response [56,57], and myelination [58,59]. These functions confer on them a key role in maintaining homeostasis, the disruption of which can lead to neuropsychiatric and neurodegenerative diseases [59,60,61,62,63,64].

**Figure 1:**
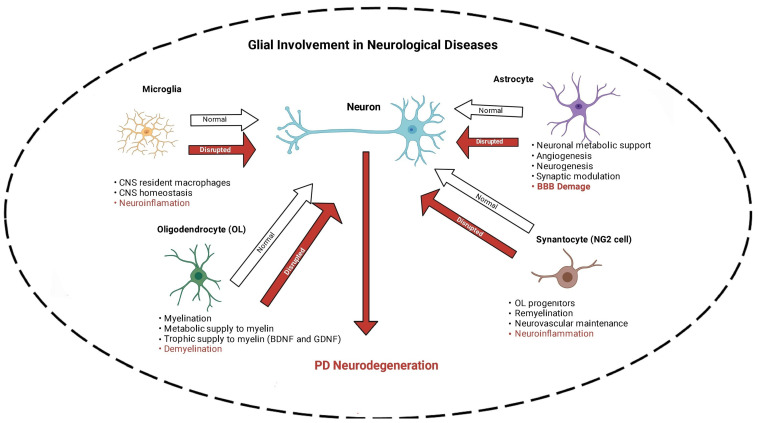
Schematic diagram of glial–neuron interaction. Glial cells are essential for maintaining neuronal function and brain homeostasis. Four types are depicted here. 1. Microglia act as innate immune cells in the brain and are critical in defense response. However, their disruption (overactivation) can lead to neuroinflammation and neurodegeneration. 2. Astrocytes perform several functions, including providing energy support for neurons, angiogenesis, neurogenesis, and synaptic modulation, disruption of which results in blood–brain barrier (BBB) damage. 3. Oligodendrocytes are pivotal for myelination and produce neurotrophic factors such as brain-derived neurotrophic factor (BDNF) and glial cell line-derived neurotrophic factor (GDNF). Dysfunction in these cells results in demyelination. 4. Synantocytes (NG2 cells) are oligodendrocyte progenitor cells and contribute to neurovascular maintenance and myelination. Their disruption leads to various damages, including neuroinflammation and neuronal death. Figure created with BioRender.com, accessed on 18 February 2024.

As mentioned above, four main subsets of glial cells (microglia, astrocytes, oligodendrocytes, and synantocytes or NG2 cells) in the central nervous system (CNS) were identified (Figure 1). Here, following the description of each glial cell type, specific role of the nAChRs in these cells in relevance to PD is discussed.

## 4. Microglia

Microglia, constituting 10–15% of all CNS cells, are an important subset of the glial cells acting as the innate immune response and play a key role in neuroinflammation, responsible for the degeneration of DAergic neurons in PD [65,66,67,68]. Indeed, microglia are considered the resident macrophages with a vital role in maintaining CNS homeostasis as they eliminate pathogens and cell residue through phagocytic activity (Figure 1). They are also crucial regulators of neurogenesis as they participate not only in the formation but also elimination of neuronal synapses and control the number of neuronal precursor cells. Furthermore, microglia mediate the autoimmune effector T-cell infiltration into the brain and hence serve as the main antigen-presenting cells in the CNS [69]. Three states of microglia consisting of resting, activated, and phagocytic were identified. At the resting stage, they are highly ramified, but when activated in response to injury or insult, they contract, assume an enlarged cell body, and proliferate. Finally, they transform into full-blown phagocytic microglia. However, if overactivated, neuroinflammatory and neurodegenerative disorders may ensue [65,67,68,70]. In general, depending on their activation phase, they are categorized as M1 microglia, which lead to neuroinflammation and neurotoxicity, and M2 microglia, which stimulate anti-inflammatory and neuroprotective effects [69,71,72].

Microglial polarization into the M1 or M2 phenotype occurs because of perturbation in the micro-environment of the microglia. Under resting physiological conditions, microglial morphology is manifested as a small cell body and very fine but highly ramified processes, which allow them to survey their local environment for signs of cellular damage or pathogens. Because of this continuous activity, it was proposed that this stage of microglia be designated as “surveilling” microglia rather than resting state [73,74,75]. When activated, the microglial cell body enlarges and processes are withdrawn or become shorter and assume a round or amoebic shape, allowing them to migrate to the site of injury and initiate phagocytic activity. Thus, amoeboid microglia, having retracted processes and a swollen cell soma, reach the site of injury and initiate the phagocytosis of harmful debris. This morphological transformation may reflect disease-specific functional cell states. Interestingly, a fourth microglial morphology, the so-called “rod-like microglial cells”, which show fewer secondary branches and narrowing of cells and soma but do not exhibit planar processes, were also identified in mice [75,76,77]. Activated microglia express a variety of receptors, such as the triggering receptor expressed on myeloid cells-2 (TREM2), low-density lipoprotein receptor-related protein 1 (LRP1), calcium-sensing receptor (CASR), and toll-like receptors 2 and 4 (TLR2 and TLR4) [78,79,80,81]. TLRs are a well-characterized family of pattern recognition receptors (PRRs), expressed by microglia and astrocytes (discussed below), that sense the pathogens or endogenous debris released by damaged cells and initiate the innate immune response. Microglial contribution to TLR signaling and CNS pathology is a subject of intense investigation as potential therapeutic targets were suggested [79,82,83]. In addition, all glial cells, including microglia, express nAChR [84], which is the emphasis of this review.

## 5. Microglia Synucleinopathies

As the name implies, synucleinopathies refer to neurodegenerative diseases due to a shared accumulation of the pathologic α-Syn protein, which, as alluded to earlier, can cause neuronal death as in PD. Although the etiology of synucleinopathies remains unknown, microglia were directly implicated in its pathogenesis. It is worth reiterating that microglia offer neuroprotection in the early stages of α-Syn accumulation; however, in chronic disease conditions, structural variations may accompany inherited faulty α-Syn, resulting in distorted microglial receptor interactions [85,86]. Thus, while microglial receptors initially enable cellular recognition and the uptake of α-Syn, in chronic disease states, the physiological functions of microglia are overwhelmed. At this stage, α-Syn processing within microglia results in neuroinflammation and neurodegeneration, which can also spread to other brain regions [85,86]. If microglia are overwhelmed, such as during a pathological state, their phenotype changes, and this helps the spread of α-Syn, causing disease progression [66,85,86]. In neurodegenerative diseases, microglial activation involves cellular metabolism dysregulation and mitochondrial damage, leading to the accumulation of reactive oxygen species (ROS), amino acids, iron, ferroptosis, and eventual inflammation and cell death [66,85,86,87]. Recently, it was revealed that circadian rhythm also plays a critical role in microglia activation and function and that the disruption of this rhythm can lead to neurodegenerative diseases [88].

Synucleinopathies are also considered a hallmark of dementia, including PD dementia (PDD) and Lewy body dementia (LBD), where the latter comprises roughly 20–30% of all dementia cases in the world and is considered the third most common form of dementia [88,89]. In Alzheimer’s disease (AD), co-morbid α-Syn pathology is present in up to 50% of cases. The cell-to-cell transmission of α-Syn aligns well with the clinical course of the disease and is now considered the underlying mechanism for neurodegenerative diseases. What may cause α-Syn misfolding in the first place is believed to involve a combination of factors, including aging, environmental factors, and exposure to toxins, which ultimately overwhelms the microglia’s ability to maintain homeostasis and leads to neurodegeneration [88,89]. Aberrant circadian rhythmicity may also lead to extensive α-Syn aggregation [90]. Interestingly, microglial gene and protein expression also follow circadian patterns, and hence, microglia’s activity (e.g., synaptic pruning) and response to an immune challenge may depend on the time of the day [91]. Thus, circadian rhythm dysfunction may precipitate neurodegenerative consequences via microglial activation and synucleinopathies.

Overall, it may be concluded that microglia play a pivotal role in the spread of α-Syn and the eventual pathophysiology of synucleinopathies, including those related to circadian rhythm dysfunction. Therefore, extensive effort is expended in elucidating the molecular mechanism(s) of microglial interactions with α-Syn in the hope of identifying novel therapeutic targets for prominent neurodegenerative diseases, including PD [85,86]. It is also worth mentioning that TLR 4 is required for the α-Syn-dependent activation of microglia [92,93]

Very recently, however, it was discovered that the α-Syn structure changes when it is phosphorylated. This change promotes its interactions with other proteins and lets it act as a brake, thus keeping in check the activity of certain neuronal circuits, making it a player in maintaining a healthy brain [94]. But why events with a relatively low frequency can accumulate over time and trigger the pathological accumulation of α-Syn, causing Lewy body dementia, is still unknown [95].

In summary, it may be suggested that the α-Syn activation of microglia via TLRs results in microglial inflammatory response and eventual destruction of dopaminergic neurons, leading to PD.

## 6. Astroglia

Astroglia or astrocytes are star-shaped cells that make up between 17% and 61% of the cells (depending on the area) in the human brain [83]. Like microglia, in response to various insults, they exhibit heterogeneous phenotypes, a process referred to as astrocyte reactivity [83,96]. Astrocytes perform myriad essential functions, including the maintenance and upholding the accuracy of brain signaling, recycling of neurotransmitters, modulation of ionic environment and providing metabolic support for the neurons, regulation of cholesterol and sphingolipid metabolism, and maintenance of BBB [89,90] (Figure 1).

Several subclasses of astrocytes were identified. The most numerous ones are protoplasmic astrocytes that have a stellate morphology and are prominent in layers II–VI of the cortical gray matter and are found in all mammals. Two other distinct subtypes are found only in primates and humans and reside in either layer I or layer VI of cortical layers. These interlaminar astrocytes express a high level of glial fibrillary astrocytic protein (GFAP) that is commonly used as markers in their identification. Moreover, astrocytes express several neurotransmitter receptors, including both α4- and α7-containing nAChRs [97,98,99,100,101].

Although astrocytes are pivotal for brain functioning, homeostasis, and detoxification [38,39,102,103], their role in PD pathogenesis is not completely known. Reports show an increase in α-Syn-immunoreactive astrocytes in postmortem human brains [104]. Moreover, in vitro studies suggest astrocytes are susceptible to dysfunction induced by α-Syn [105] or aminochrome [106,107,108,109,110]. As mentioned, α-Syn is an unfolded protein that accumulates in Lewy bodies, a hallmark of PD [111]. Aminochrome, on the other hand, is an endogenous DA neurotoxin [11]. Compared to microglia, astrocytes are not well equipped with receptors that can recognize pathogens. However, they can become reactive and release inflammatory mediators when activated by polarized microglia, hence leading to inflammation [69,112,113,114]. Thus, astrocytes can amplify proinflammatory signals released by microglia and contribute to neuronal degeneration, suggesting synergistic collusion [105,106,107] (Figure 1). Interestingly, like microglia, the α-Syn activation of astrocytes is dependent on the presence of TLR4 in these cells [92,93]. Moreover, an intimate interaction between astrocytes and neurons, as well as between astrocytes and microglia, referred to as crosstalk, was recently highlighted [83,97]. As our knowledge of such crosstalk expands, novel interventions for neurodegenerative diseases, including PD, are anticipated [83,97,115]. Furthermore, because of the nAChRs’ presence in these cells and their known functional role (discussed below), specific targets may be suggested [98,99,101,116].

## 7. Oligodendrocytes

Oligodendrocytes (OLs), once considered the static glue, are not only the myelinating cells of the CNS but are also plastic and adaptive to changes in CNS [110] (Figure 1). Four phases in the life cycle of OLs were identified: (1) OL precursor cells (OPCs) giving rise to birth, proliferation, and migration of OLS. Remarkably, OPCs by themselves constitute a subclass (fourth subset) of glial cells that are described in detail below; (2) morphological differentiation, characterized by an elaborate network of processes; (3) the generation of compact myelin and the ensheathment around target axons; and (4) the metabolic and trophic support of the encased axon [117]. It is of relevance to note that in the peripheral nervous system, neuroglia that are equivalent to OLs are called Schwann cells [118,119].

The importance of OLs in the pathogenesis of PD was also verified [120,121] (Figure 1). A single-core human transcriptomic atlas for the substantia nigra (SN) revealed that distinct neuropsychiatric disorders associated with neuron-specific genes converge on shared loci within OLs and OPCs [120]. These cells represent 75% of all glial cells in the adult CNS. In addition to axonal myelination, OLs control extracellular potassium concentration and, as mentioned above, provide metabolic and trophic supply to myelin, secrete glial and brain-derived neurotrophic factors (GDNF and BDNF), and modulate the axonal growth [117,122,123], all of which highlight their importance in the functioning of CNS. Like microglia and astrocytes, OLs also express TLRs, which are considered of significant importance in myelin formation [58,124,125]. Importantly and of direct relevance to our discussion, the dysregulation of these glial cells, which contain nAChRs, can contribute to the pathogenesis of PD [121].

## 8. Synantocytes (NG2 Cells)

The fourth subset of glial cells in CNS are synantocytes or neuron glial 2, or nerve/glial antigen 2 (NG2) cells. These cells, also referred to as OPCs, are identified primarily by the presence of two key markers: the chondroitin sulfate proteoglycan NG2 and the platelet-derived growth factor receptor alpha (PDGFRα) [117,126]. These cells display a combination of features, including (i) an almost uniform presence in both cell body and myelinated axons; (ii) a complex stellate morphology; (iii) an intimate association with cell bodies and dendrites of neurons; (iv) the capacity for continued proliferation in the adult brain; and (v) a latent ability to generate astrocytes and neurons to be recruited to the lesioned area [117,126,127].

While the main role ascribed to NG2 cells was originally that of progenitors for OLs, it was later discovered that they perform a variety of functions in the brain, including the performance of potential roles in demyelinating and neurodegenerative diseases such as multiple sclerosis and AD, as well as traumatic brain injury, glioma, epilepsy, and electroconvulsive therapy for depression [128,129]. Additionally, in pathological conditions, they were recognized as an early marker of pericyte activation and were suspected of playing a role in experimental autoimmune encephalomyelitis (EAE), a condition where BBB permeability is increased, and neuroinflammation ensues [130]. It was suggested that NG2 cells exert their effects via the stimulation of reactive T cells and by controlling IL-12 expression [130].

More recently, it was suggested that NG2 cells play a critical role in the modulation of neuroinflammation [131] and neurovascular unit formation during development [123] (Figure 1). Although no TLRs have yet been identified in these cells, their importance in angiogenesis and oligodendrogenesis following acute ischemic stroke was reported [132]. Importantly, their ability to receive synapses from neurons and affect neuronal plasticity and behavior and their containment of nAChRs [133,134] suggest their potential use in specific therapeutic interventions.

## 9. Nicotine

Nicotine, the primary psychoactive agent in tobacco leaves, is highly addictive and has, therefore, led to the global use of tobacco, where it is estimated that over one billion people smoke. The severe consequences of smoking on almost every organ of the body and the manifestation of numerous diseases, which result in nearly 500,000 deaths in the US alone and nearly 8 million people worldwide, are staggering statistics. Diseases associated with smoking include a variety of cancers, especially that of the lung, but also cancers of the voice box, throat and mouth, stomach, kidney, esophagus, pancreas, bladder, liver, colon and rectum, cervix, and acute myeloid leukemia [135]. Additionally, the risks of stroke, heart disease, and chronic obstructive pulmonary disease (COPD), including emphysema and chronic bronchitis, diabetes, certain eye diseases, tuberculosis, and immune dysfunction, including rheumatoid arthritis, are well established [135]. However, nicotine by itself may have many therapeutic potentials, including neuroprotection (discussed below). Specifically, as our understanding of the mechanism of action of nicotine expands, more selective therapeutic targets become available. The following provides an up-to-date summary of the current knowledge on nAChRs, with specific implications in PD.

## 10. nAChRs

The action of nicotine is mediated via nAChRs, which act directly to open a channel, allowing for the influx of sodium (Na^+^) and calcium (Ca²^+^) [136,137,138,139]. The nAChRs are pentamers composed of different subunits such as alpha (α), beta (β), or delta (δ). To date, overall, 16 homologous mammalian nAChR subunits have been identified [136,138]. Many different nAChR subtypes due to subunit combination form in different areas, such as the neuromuscular junction, autonomic ganglia, and CNS [136,137,138,140]. However, the subunit structures of these receptors are different depending on the area. For example, only the neuromuscular receptors contain the delta subunit, whereas the autonomic ganglia and CNS nAChRs contain only alpha and beta subunits, albeit in different combinations [136,137,138,140,141]. In the brain, the predominant subtypes consist of alpha4 and beta2 or the homomeric alpha7 subtypes [136,137,138,140,141,142].

The α7 subtype of nicotinic receptors (α7nAChRs) is one of the most abundant nicotinic receptor subtypes in the CNS, and both neurons and nonneuronal cells express it [136,140,141,143]. When activated, α7nAChRs allow the flow of cations, promoting cellular responses, including the modulation of the PI3K/Akt signaling cascade. This results in the anti-apoptotic stimulation of molecules of the Bcl-2 family, Bcl-2 and Bcl-xl, and the reduction of proapoptotic molecules, hence promoting cell survival [144]. α7nAChR is also a key protein in the cholinergic anti-inflammatory pathway (discussed in detail below) that links the nervous and the immune systems [145].

More recently, the potential metabotropic signaling responses by α7 nAChRs through heterotrimeric G proteins in both neuronal and immune cells was suggested [146]. Furthermore, and of relevance to our discussion below, not only non-ionic signaling mechanisms via nAChRs were demonstrated in immune cells, but some nAChRs may also be activated by endogenous ligands other than ACh [147].

Nonneuronal nAChRs also exist and are expressed in lung epithelial, endothelial, and fibroblast cells, as well as in muscles. Chronic nicotine exposure differentially affects the expression of different receptor subtypes, such as an increase in the α5 subunit of nAChRs in epithelial cells and a decrease in the α3 subunit in fibroblast cells [148]. Further nicotinic receptor subtype distinctions are evident in their physiological roles and central distribution. Thus, alpha7 or the low-affinity subtype, which are most abundant in the hippocampus, play a prominent role in neuronal survival and growth and are involved in various cognitive functions, including attentional processes [35]. These receptors are also implicated in pain modulation [149]. On the other hand, the high affinity α4β2 subtype is mostly expressed in the mesolimbic reward system and is associated with addictive behavior, whereas its presence in the nigrostriatal pathway involves locomotor activity and antinociception [150].

It is now evident that nAChRs not only play an important role in neuronal function and the addiction to nicotine [151,152] but may also serve as targets for therapeutic intervention in various neuropsychiatric/neurodegenerative disorders, including PD [25,153,154], depression [153,155,156], obsessive–compulsive disorder [157,158], ADHD [159], Tourette syndrome [160,161,162], mild cognitive impairment or Alzheimer’s disease [163,164,165,166], ischemia [167], catalepsy [168], schizophrenia [4,166,169], pain [147,170], energy balance [171,172], autoimmune disorders [173], and even sleep–wake cycle dysregulation [174]. These receptors are also expressed abundantly in a variety of immune cells, including B cells, T cells, macrophages, and microglia, and are believed to contribute to the anti-inflammatory effects of nicotine [175,176,177,178]. Indeed, nicotine was shown to inhibit the pro-inflammatory cytokines such as tumor necrosis factor- α (TNF-α), IL-1, and IL-6 without affecting the anti-inflammatory cytokines such as IL-10 [177,178,179,180]. This effect of nicotine, in addition to its interaction with ACE2 via nicotinic receptors, has led to the suggestion of a potential role of selective nicotinic receptors in interfering with the SARS-CoV-2 virus entry, hence improving COVID-19 conditions [148,181]. In relation to cancers such as lung cancer, there is an increase in the expression of nAChRs in this organ, which was associated with cell proliferation, angiogenesis, epithelial-to-mesenchymal cell transition, and the prevention of apoptosis [182]. In relation to diabetes, a role for nAChRs in glucose tolerance, the release of glucoregulatory hormones, and sensitivity to insulin was recently suggested [172]. Therefore, the modulation of the nicotinic receptors by appropriate concentrations of nicotine may be exploited for a plethora of therapeutic purposes.

## 11. Nicotine for PD

It is now evident that basal ganglia’s normal function depends on the equilibrium between the striatal cholinergic and midbrain dopaminergic systems [183,184,185]. Acetylcholine (ACh), via interaction with nicotinic receptors, regulates striatal DA release [25,154]. Moreover, the impairments in DA release evident in animal models of PD (e.g., 6-OHDA lesioned rodents) appear to be exacerbated by a loss of nAChRs, which suggests that DAergic imbalance may be ameliorated by nicotinic agonists, hence their usefulness in PD. Indeed, several in vitro and in vivo studies in rodents and primates, including genetically modified mice, verified the protective effects of nicotine against neuronal damage induced by 6-OHDA, MPTP, rotenone, paraquat, methamphetamine, glutamate, and β-amyloid [3]. Nicotine also protects against salsolinol-induced toxicity in SH-SY5Y cells [186]. Salsolinol, an endogenous product of aldehyde and DA condensation, is frequently used to induce selective toxicity to dopaminergic neurons. SH-SY5Y cells, derived from human neuroblastoma, are commonly used as a cellular model of dopaminergic neurons to investigate novel treatments for PD [187,188]. It is now evident that both alpha4-beta2 and alpha7 nicotinic receptors are involved in the protective effects of nicotine [3,186,189]. Similarly, the damage inflicted by aminochrome, a neurotoxic molecule derived from DA oxidation, on RCSN-3 cells (derived from substantia nigra of adult rats) could also be prevented by nicotine [190]. More recently, the protective effects of nicotine against the toxicity induced by manganese and iron in SH-SY5Y cells with implications for PD were reported [187,188]. Additionally, it was shown in vitro that nicotine protects PC12 neural cells against toxicity induced by1-methyl-4-phenylpyridinium ion (MPP+) via activation of alpha7 nAChR/PI3K/Trx-1 signaling and suppression of endoplasmic reticulum stress [191,192]. This finding was recently complemented by the findings that nicotine also alleviates the MPTP-induced damage to the nigrostriatal pathway via the modulation of JNK and ERK signaling pathways in a mouse model of PD [193]. Moreover, using D-line α-Syn transgenic mice and a humanized neuronal model of synucleinopathies, it was revealed that nicotine, via the activation of α4β2 nicotinic receptors, attenuates α-Syn-provoked neuropathology [194]. In a similar in vivo study, it was shown that nicotine attenuates motor deficits in an α-Syn PD model [195]. Thus, manipulating both α4β2 and α7nAChRs may effectively mitigate synucleinopathies [178,194,196].

The fibril-destabilizing and anti-fibrillogenic activities of nicotine, in addition to its ability to promote the clearance of α-Syn, may be of critical importance in its inhibition of Lewy bodies [197,198,199,200,201,202,203]. As alluded to earlier, the accumulation of Lewy bodies, composed primarily of α-Syn, is a hallmark of PD pathology. Indeed, it is believed that synucleinopathies not only contribute to movement disorders but also to cognitive and social impairment associated with PD [203,204,205]. In further support of the contention that targeting nAChRs may be a novel therapeutic strategy for PD treatment [99,206], it was recently reported that nicotine’s prevention of synucleinopathies or α-Syn toxicity may be due to its interaction with α7nAChRs and inhibition of apoptosis as well as interaction with synaptic vesicle glycoprotein [201,207].

## 12. Mode of Nicotine Administration as a Critical Factor in PD

The well-established inverse relationship between smoking and PD and documented neuroprotective effects of nicotine prompted several clinical trials with nicotine patches in PD [208]. However, no apparent benefit was noted with such a mode of nicotine administration [208]. This lack of response to nicotine patches was likely due to the steady release of nicotine and prolonged nicotinic receptor desensitization [3,185]. Although a nicotine patch, by maintaining a steady plasma concentration of nicotine, may be an effective intervention for smoking cessation as the desensitization of the central nicotinic receptors in critical brain reward circuitry may help ameliorate the withdrawal effects of nicotine, it is unlikely to be therapeutically efficacious for PD. This contention is underscored by the fact that a pulsatile stimulation of nicotinic receptors, like that experienced by smokers, is necessary to re-stimulate the nicotinic receptors and, hence, provide neuroprotection in PD [3,208]. It is also of utmost importance to distinguish between pure nicotine and nicotine derived from burning tobacco leaves, where the latter contains xenobiotics, including carcinogens and other toxins. Moreover, nicotine by itself may not only be effective in ameliorating PD symptoms and retard the progression of the disease but may also counter L-dopa-induced dyskinesia [3,185].

It is worth mentioning that current efforts to develop selective nicotinic receptor modulators or agonists that could be similarly effective but without addictive properties would be of significant therapeutic triumph [3,209,210,211].

## 13. nAChR–Microglia and Gut–Brain Axis

**Figure 2:**
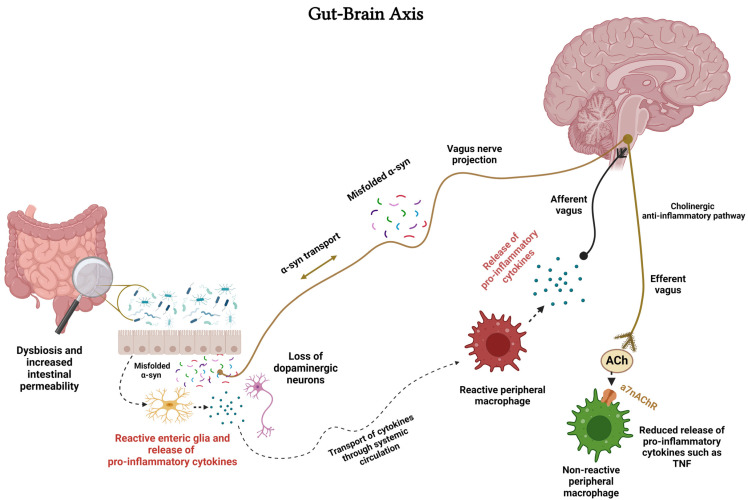
Schematic diagram of the gut–brain axis and cholinergic anti-inflammatory pathway. Dysbiosis and increased intestinal permeability cause reactivity of enteric nervous system, including enteric glial cells. This results in the release of inflammatory cytokines and misfolded α-synuclein (α-syn), which can cause neuronal damage. As a compensatory response, the cholinergic anti-inflammatory pathway is activated to reduce the release of pro-inflammatory cytokines from the peripheral macrophages. ACh = acetylcholine, TNF = tissue necrosis factor. Figure created with BioRender.com.

Microglia, as discussed in detail above, are resident macrophages of CNS. Macrophages refer to cells that serve as vital defenders in response to various stimuli or invading pathogens. During the peripheral immune challenge, inflammatory cytokines can signal the brain for activation of the immunomodulatory mechanism via the vagus nerve (Figure 2). This involves the activation of the cholinergic anti-inflammatory pathway, the release of ACh from the vagus nerve, and the activation of α7nAChRs on peripheral macrophages to restore homeostasis [190,191,192,193,212,213] (Figure 2).

Moreover, the cholinergic anti-inflammatory pathway functions as an interface between the brain and the immune system [212,213,214]. It is noteworthy that the vagus nerve was also implicated in brain–immune system interaction as it connects the brain to the gut and vice versa; hence, it is an integral part of the gut–brain axis (GBA) (Figure 2). GBA, considered to play a central role in many diseases, including neurodegenerative/neuropsychiatric disorders, is influenced by the gut microbiota (GM) via a variety of molecules and neurotransmitters, including short-chain fatty acids (SCFAs) [214,215,216,217]. GBA communication occurs through several pathways, including the immune system, the enteric nervous system (ENS, a complex of neuronal and glial networks controlling the function of the gut), microbial metabolites, and the vagus nerve [218]. GM may also influence neural function via its interaction with microglia and astroglia [218]. Thus, neuroinflammation and its devastating consequences can arise from an imbalance in GM, referred to as dysbiosis or disruption in any component of the GBA [219,220].

Regarding ENS, it is important to note that the status of the gut is communicated to the brain via sensory neurons arising from the dorsal root, nodose ganglia, neurons of the autonomic nervous system, and immune cells of the gut. The Vagus nerve is a critical component in GBA as it coordinates the immune system’s response to bacteria, pathogens, or toxins. In this regard, the afferent vagus nerve is considered the main retrograde signaling system, bringing information from the gut to the brain. On the other hand, the efferent vagus nerve, containing the cholinergic anti-inflammatory activity, helps control TNF-α and other cytokines secreted by macrophages in the gut [221]. One common element to all GBA components is the expression of nAChRs that serve myriad roles in this axis (Figure 2). These roles include fast synaptic transmission mediation between autonomic pre- and postganglionic neurons, modulating the release of neurotransmitters from the enteric and peripheral sensory neurons, and controlling the release of cytokines from the immune cells [216].

The involvement of GM in the pathogenesis of PD via immunological, neuroendocrine, and direct neural mechanisms is well acknowledged [222,223]. The sequence of events is believed to start with GM dysbiosis, which triggers the eventual loss of DAergic neurons via mitochondrial dysfunction and systemic inflammation (Figure 2). Moreover, GM dysbiosis results in the activation of enteric neurons and enteric glial cells, leading to the aggregation of α-Syn. Curiously, α-Syn is also synthesized by the sensory cells of the gut mucosa and can be transported to the CNS via the vagus nerve [198,199] (Figure 2). Indeed, according to Braak’s hypothesis, α-Syn misfolding begins in the gut and spreads “prion-like” via the vagus nerve into the lower brainstem and ultimately to the midbrain, leading to PD [221]. Because of its enteroendocrine cell-mediated interaction with GM, the vagus nerve may also provide a pathway whereby the gut microorganisms may influence the feeding behavior of the host, thus suggesting a role for the vagus nerve in appetite control, the development of obesity, and diabetes as well [224].

**Figure 3:**
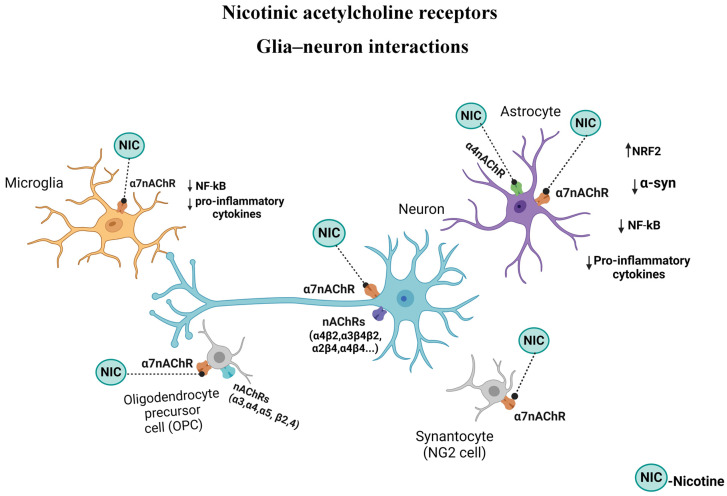
Schematic diagram of nicotinic acetylcholine receptors (nAChRs) on glial and neuronal cells and consequence of activation by nicotine. Activation of selective nAChRs in microglia and astrocytes results in an anti-inflammatory response. In oligodendrocytes (OLs), stimulation of nAChRs may result in remyelination and, in NG2 cells, to anti-inflammatory response, although the latter has yet to be verified. NRF2 = nuclear factor erythroid 2-related factor, α-syn = αsynuclein, NF-kB = nuclear factor kappa-light-chain-enhancer of activated B cells. Figure created with BioRender.com.

Because nAChRs play a critical role in the function of GBA, their manipulation along this axis and in microglia may provide novel interventions in neuropsychiatric/neurodegenerative diseases (Figure 3). As mentioned above, sufficient data are now available to justify the use of pulsatile nicotine in PD. This contention is further strengthened by the findings that nicotine may modulate the release of inflammatory cytokines and the expression of TLRs [225,226]. Indeed, recent findings indicate the inhibition of TLRs by cotinine, a metabolite of nicotine via α7nAChR [35].

As our understanding of the intricate interactions between GM, microglia, and, specifically, the role of nAChRs in these scenarios expands, more therapeutic targets may become available. In this regard, the recent discovery of the partial duplication of α7nAChR, known as dup α7, is gaining attention as a key player in macrophage polarization and neuroinflammation [214]. Additionally, nAChRs may not only provide a therapeutic target but may also function as a diagnostic tool. For example, it was proposed that the imaging of microglia and nAChRs in the living brain may be able to identify the stage of AD [227].

## 14. nAChR–Astroglia

Astroglia, as mentioned above, also express nAChRs. Indeed, the role and manipulation of astrocytes nAChRs in neurodegenerative diseases such as AD and PD are well documented (Figure 3). Thus, the stimulation of these receptors by nicotine or other agonists may suppress synaptotoxicity, amyloidosis, oxidative stress, and neuroinflammation, all of which are strongly implicated in AD and PD [99,228]. Specifically, it was shown that the manipulation of α7nAChRs suppressed reactive astrogliosis; the release of cytokines such as IL-6, IL-1β, and TNF- α; gliotransmitters such as ATP and glutamate; and potentially, Aβ plaque deposition [229,230]. Cholinergic projection arising from the nucleus basalis of Meynert (NBM), projecting to the entire cortical layer, the olfactory tubercle, hippocampus, and the amygdala, is believed to be critical in the regulation of attention and arousal, both integral components of learning and memory. A role for NBM in pain modulation was also recently reported [231]. Interestingly, astrocytic α7nAChRs, by maintaining Ca^2+^ homeostasis, are considered essential for synaptic plasticity across cortical and hippocampal regions [230,232]. Moreover, the stimulation of these receptors on astroglia leads to the inhibition of the NF-κB and activation of the Nrf2 pathways, conferring anti-inflammatory effects on astroglia [233,234]. Additionally, the potential diagnostic use of α7nAChRs as markers of reactive astrogliosis, at least in AD, was proposed [230]. Using astrocyte-specific manipulations, it was shown that potentiating astrocyte Ca^2+^ signaling in the hippocampal CA1 region enhances temporal association, deemed essential for memory formation, whereas the attenuation of astrocyte Ca^2+^ signaling imparts the opposite effect [101]. Curiously, these effects were mediated primarily by α4 containing nAChR subunit on the astrocyte. The same subunit was implicated in the cognitive enhancement effects of nicotine as evidenced by fear conditioning tests and object–trace–odor paired-associate tasks in mice [94]. Recently, the presence of other subunits containing nAChR, including α4 in the ventral tegmental area (VTA), an area implicated in reward circuitry, was verified, suggesting a potential role for these astrocytic nAChRs in addictive behavior [100]. Thus, the significance of both α4 and α7 containing nAChRs in astrocyte functions is evident (Figure 3). Additionally, like in microglia, astrocytic TRLs also play a pivotal role in sterile inflammation and the pathogenesis of neurodegenerative diseases [93]. Thus, further elucidation of the specific role of nAChR subtypes in astroglial TRL functioning may provide more selective and targeted intervention in PD.

In summary, although more studies are required to investigate the potential role of specific astroglial nAChRs in neuropsychiatric/neurodegenerative disorders, at this juncture, it may be suggested that activating α7nAChRs in these cells may confer neuroprotection by decreasing inflammation and oxidative stress, whereas the stimulation of both α7 and α4 containing nAChRs in astrocytes may be of therapeutic potential in cognitive improvements [93,100,101,230,233,234].

## 15. nAChR–Oligodendrocyte (OL)

OLs play a substantial role in CNS by forming a myelin sheath, which is critical in accelerating nerve conduction and maintaining the neuronal signaling process. Demyelination, due to the death of OLs and a loss of myelin sheaths, may lead to clinical disorders such as stroke, dementia, multiple sclerosis (MS), schizophrenia, and chronic cerebral hypoperfusion [123]. Increased myelin formation may be brought about by enhancing the signaling between neurons and OLs [235,236], which can lead to OPC differentiation, proliferation, and maturation, as well as neural pathway integrations [237,238]. Enhanced neuronal activity may be induced directly or indirectly by various techniques such as chemogenetics, optogenetics, sensory stimulation, transcranial stimulation [239,240], or potentially via pharmacological manipulation. Regarding the latter, and of relevance to the current topic, ACh involvement in myelin formation and the presence of both muscarinic and nAChRs in OLs was verified [123,241,242]. In fact, demyelination disorders are associated with defects in cholinergic anti-inflammatory signaling pathways mediated by α7nAChRs (Figure 3). Interestingly, whereas nicotinic stimulation boosts OPC maturation and myelin regeneration, muscarinic stimulation has the opposite effect in that OPC differentiation and myelin regeneration are retarded [21,214]. Nonetheless, the specific impact of nAChR signaling in relation to OL functioning in general and to PD in particular has yet to be fully revealed [123]. Moreover, since OLs also express TLRs, the elucidation of nAChRs’ interaction with TLRs in these cells may offer novel therapeutic targets not only in demyelination diseases but also in PD [121].

## 16. nAChRs–NG2 Cells

NG2-glia are heterogeneous glial cells with distinct properties whose dysfunction can affect neuronal plasticity, leading to neurological and behavioral consequences [134]. Regarding the latter, the role of NG2 cells in stress-related mental disorders was recently reviewed [243]. It was concluded that dissecting the complex biology of NG2 glial cells and delineating their causal role in stress-related psychopathologies and stress response may provide novel interventions in such behavioral disorders [243].

NG2 cells alter their function in response to insults, including viral encephalopathy, rendering them potential targets in preventing viral infection-induced epilepsy [244]. Although it was well established that neurons synapse on NG2 cells and have a modulatory role in their development and regeneration, very recently, it was revealed that NG2 cells may themselves act as neural progenitor cells. This finding, which was reported in the cortex of adult mice [245], if verified in the human neuronal system, can have a wide implication in providing regenerative interventions for neurodegenerative diseases [245].

As mentioned earlier, NG2 cells may play a critical role in the modulation of neuroinflammation [228]. NG2-positive cells co-expressing ionized calcium-binding adaptor molecule 1 (Iba1) were identified in SNpc and the striatum of a rat model of PD [246]. This finding, together with the observation that ablation of NG2 cells exacerbates DAergic neuronal cell loss in a mouse model of PD, suggests that NG2 cells may act as negative regulators of neuroinflammation [228]. Moreover, a critical role of NG2 cells in protecting the neurovascular unit and angiogenesis after acute ischemic stroke was recently suggested [123] (Figure 3). It was proposed that exosomes derived from dental pulp stem cells may promote NG2-glia proliferation and differentiation and, hence, reduce tissue damage due to acute ischemic stroke [132]. Exosomes are small extracellular vesicles secreted by various stem cells and are potent mediators of intercellular communication and tissue repair [166,247]. Recently, clinical applications of exosomes in general surgery, neurosurgery, cardiothoracic surgery, orthopedic surgery, head and neck surgery, plastic surgery, acute skin wound healing, urology, ophthalmology, and obstetrics and gynecology, and other diseases induced by ischemia, inflammation, or cancer were suggested [248,249].

Regarding neuroinflammation, distinct roles for TLRs, as well as nAChRs in neuroinflammatory diseases, including PD, are well documented (see above). Although no TLRs in NG2 cells have yet been identified, these cells’ possession of α7nAChRs is confirmed [124,125] (Figure 3). Moreover, a recent report implicates striatal NG2-glia in L-dopa-induced dyskinesia [250]. Curiously, in a mouse model of preeclampsia, it was reported that nicotine has favorable modifications of the trophoblast-derived exosomes [251]. Exosomes, by carrying genetic material such as microRNAs, can regulate cell function and may not only serve as a biomarker of disease state but may also be of therapeutic potential [247,248,249]. Therefore, it is of high interest and applicability to determine whether nAChRs may have a role in exosome production and/or effect, particularly in relation to neurodegenerative diseases.

Thus, the further elucidation of the potential role of nAChRs, as well as the interaction of these receptors with TLRRs in NG2 glial cells, may provide novel intervention in neurodegenerative and/or neuropsychiatric diseases, including PD.

## 17. Conclusions

In summary, based on pre-clinical and epidemiological data, nicotine is a potential drug for PD. Although the significance of neuronal nAChRs in the action of nicotine and other modulators of these receptors is well documented, only relatively recently have the significance of nonneuronal nAChRs, particularly those expressed in glial cells, emerged. Given the significance of the glial cells in myriad synaptic functions and their eventual role in neuroinflammation, novel therapeutics targeting these receptors are envisioned. This contention is strengthened by emerging interactions between nAChRs and TLRs, where the latter plays a critical role in neuroinflammatory diseases. Furthermore, recent evidence of glial influence on GBA and the potential manipulation of this axis by nAChRs warrant further investigation. It is anticipated that such investigations will culminate in novel interventions in neuropsychiatric/neurodegenerative diseases, including PD.
